# INTERVENING IN THE LONGEVITY NETWORK

**DOI:** 10.1093/geroni/igz038.2293

**Published:** 2019-11-08

**Authors:** Alexander Mendenhall, George L Sutphin

**Affiliations:** 1 University of Washington, Seattle, Washington, United States; 2 University of Arizona, Tucson, Arizona, United States

## Abstract

This session will focus on interventions to delay biological aging with the goal of in increasing lifespan and healthspan.

